# Views of Catholic Priests Regarding Causes, Treatments and Psychosocial Consequences of Schizophrenia and Depression: A Comparative Study in Italy

**DOI:** 10.1007/s10943-020-01138-w

**Published:** 2020-12-02

**Authors:** Lorenza Magliano, Giulia Citarelli, Gaetana Affuso

**Affiliations:** Department of Psychology, University of Campania “Luigi Vanvitelli”, Viale Ellittico 31, 81100 Caserta, Italy

**Keywords:** Schizophrenia, Depression, Religion, Priests, Causal beliefs, Attitudes, Stigma

## Abstract

This study explored views of Catholic priests about schizophrenia and depression in Italy. Participants completed a questionnaire on their views about either schizophrenia (*N* = 282) or depression (*N* = 277). The depression group was surer than the schizophrenia group that: the disorder was due to psychosocial causes; curable; non-requiring long-term pharmacotherapy; the persons with depression could participate in religious activities. The older priests were more convinced than the younger priests that: the prayer and long-term pharmacotherapy are useful; the persons with mental disorders had affective difficulties, are recognizable and kept at distance. Priests should receive education on stigma in mental disorders, particularly schizophrenia.

## Introduction

Worldwide, around five billions of people adhere to a religion, mostly Christianity (2.2 billions) and Islam (1.6 billion; Hackett 2011). Like any member of society, some of these believers may suffer from a mental disorder (MD). Research reveals that persons with MDs mostly consider religion to be helpful in dealing with their own mental health problems (Koenig 2009; Mohr et al. 2012; Weber and Pargament 2014). In a US survey of 406 persons with long-term MDs, 80% of the sample used religion to cope with their symptoms (Tepper et al. 2001). In another US study of 157 persons with severe MDs, 50% valued religious practice as useful for their mental status and as an alternative therapy to medical treatments (Russinova et al. 2002). In a study on 276 people with schizophrenia in Switzerland, Canada and North Carolina (USA), 87% of participants acknowledged the beneficial effects of religion on their mental health, while 13% stated that religion was distressing (Mohr et al. 2012). The relevance of religion from the perspective of persons with MDs is supported by studies showing that religious practice is associated with improvement of psychiatric symptoms. A systematic review of 43 studies carried out in the period 1990–2010 (Bonelli and Koenig 2013) found that religion/spirituality was associated with less severe psychiatric symptoms in 72% of cases. In particular, religious practice was found to be associated with less severe symptoms in 79% of studies on depression and 40% of studies on schizophrenia. Another study reported increased probability transient psychotic states associated with intense religious experiences (Linden et al. 2010). Adherence to religions among people with MDs is positively valued by mental health professionals (Bonelli and Koenig 2013; Vasegh et al. 2012) In a US study, 76% of participating psychiatrists acknowledged religion/spirituality as having beneficial effects on patients’ mental state (Curlin et al. 2007). In another US study, 82% of participating psychologists thought religion more beneficial than harmful to mental health (Delaney et al. 2007).

In many religions, spiritual leaders also serve as advisors in non-religious matters, such as health problems. However, the role of spiritual leaders in these matters may differ according to religion. Priests are more likely to be contacted for mental health problems than psychiatrists and psychologists combined (Hohmann and Larson 1993). Compared to psychiatrists and psychologists, priests sometimes are perceived as more capable of warmth, caring, professionalism and listening skills (Milstein et al. 2003, cited by Ellison et al. 2006). In a study on persons with MDs (Wang et al. 2003), 25% had sought a priest’s advice and of these, 25% had a severe MD. Moreover, 23% of the sample had contacted priests before general practitioners, psychiatrists or other specialists and 56% had contacted only priests in the previous year. Another study exploring the ability of psychologists and rabbis to identify schizophrenia in a case-vignette setting found no significant difference in the diagnostic skills among these groups. Compared to psychologists, rabbis were more skeptical regarding the usefulness of psychotropic drugs, and they more tended to interpret schizophrenia as due to religious causes (Milstein et al. 2000). It should be also mentioned that some persons with MDs avoid seeking treatments from physicians and therapists and prefer to seek the help of priests instead. Sometimes this is because persons with MDs are not trusting of medical systems or medication or fearful of them (Carey and Del Medico 2013). The involvement of priests as consultants in mental health issues varies according to the type of MD. In a US study on 1231 lay people, 78.8% of respondents viewed clergy as helpers in the case of schizophrenia and 91.0% in the case of depression (Ellison et al. 2006).

Given the relevance of priests’ advice for believers with MDs, it is worthwhile to investigate priests’ beliefs regarding persons with these disorders. However, only a few studies have specifically examined this issue. In a survey among 115 members of the Catholic clergy in Lebanon (Aramouny et al. 2020), 85% of the sample indicated traumatic experiences and 75% named stress among the causes of MD. Chemical imbalance was reported by 70% of respondents, and heredity by 48% of them. Supernatural causes, such as diabolical possession, were reported by 22% of the sample. Twenty-six percent believed that prayer was more important than drugs as treatment of MDs, although 80% of the sample valued drugs as helpful to cope with daily stress. In this study, levels of authoritarianism were found to be higher among clergy members who were surer of the supernatural origin of MDs and lower among clergy members with previous contacts with persons with MDs. A study conducted in Sydney on the views regarding causes of MD and effects of drugs (Youssef and Deane 2003) among 170 Arabic-speaking clerics found that most of the sample viewed drug and alcohol addiction (93.5%) and psychosocial factors (stress 93%, trauma 91%) as very/somewhat important causes of MD, while biological factors and religious causes were less frequently endorsed (heredity 76.5% and chemical imbalance 64.1%; spiritual poverty 84.7% and possession 57.1%). Prayer/spirituality was considered more useful than drugs by 43% of the sample.

Conversely, in a study of 1031 US Methodist pastors (Lafuze et al. 2002), biological causes were considered more important than psychosocial causes, religious causes and fate. Moreover, 53% of the sample considered persons with MD more dangerous than the others. None of the above-mentioned studies has been carried out in Europe, where 72% of the population is Christian and 48% are Catholic (Eurobarometer 2012). Furthermore, most studies have explored clergy beliefs regarding MDs generically, which could make the results not fully representative of priests’ beliefs about MDs with high stigma, such as schizophrenia. Finally, most studies concerned clergy views of causes and treatments of MDs, while almost no study specifically explored priests’ attitudes about persons with MDs participating in religious celebrations and group activities.

In the period 2017–2019, we conducted a study on views regarding persons with MD in a large sample of Catholic priests in Italy, a country where 74% of people (about 45 millions) claims to be Catholic (Ipsos Game Changers 2017). Participants were invited to read either a random description of schizophrenia or depression and then to complete a questionnaire on “Persons with a condition like that reported in the description”. Data were collected face-to-face in the parish churches, using a validated questionnaire (Magliano et al. 2017a, 2018) that was adapted to the survey context and re-validated. The views measured were about causal belief, recovery, usefulness of bio-psychological treatments and religious practices, the need for long-term drug treatments, insight, recognizability and affective difficulties of persons with MDs, dangerousness and social distance from persons with MDs, and persons with MDs participating in parish activities and religious ceremonies. In this paper, we report the priests’ views on the variables mentioned above and test whether the priests’ views varied in relation to the type of described disorder. We predicted that compared to priests who completed the questionnaire after reading a clinical description of depression, those who completed the same questionnaire after reading a clinical description of schizophrenia: would place more relevance on biological factors and less relevance on psychosocial factors in the development of the disorders, would give more relevance to biomedical treatments, have more negative attitudes toward persons with MDs, and would be more skeptical regarding the persons with schizophrenia participating in religious activities and celebrations. We also tested whether priests’ views of persons with MDs would be influenced by the priest’s age. We expected that compared to the younger priests, the older priests would give less relevance to psychosocial factors as causes of MDs; are more convinced of the need of long-term biomedical treatments; have more negative attitudes toward persons with MDs, and are more uncertain about the participation of persons with MDs in religious activities and celebrations.

## Methods

### Study Design

The study was approved by the Research Ethical Board of the Department of Psychology of the University of Campania “Luigi Vanvitelli” (n. 22/2016 Department Board 6/12/16, and 16/2019 Department Board 14/5/2019) and conducted in accordance with the Helsinki Declaration. Data were collected from February 2017 to December 2019. The study was carried out on Catholic priests in parishes included in six religious jurisdictions located in Southern Italy and serving 181 populated areas. Jurisdictions were selected opportunistically considering the geographical closeness to the Department of Psychology. Parishes were identified by referring to the official websites of religious jurisdictions. Parish priests were contacted personally or by phone by a researcher and invited to participate in a survey regarding their views about persons with MDs. Eligible participants were asked to consent to participate by providing them with detailed information about the purpose of the study and how the data would be collected and processed (anonymity, aggregated data analysis, right of the participant to stop completing the questionnaire at any time and leave the questionnaire blank). Informed consent was sought in writing. However, several priests preferred to participate by giving verbal consent. Participants were asked to complete the priest version of the Opinions on mental illness Questionnaire (OQ-priest) after reading a randomly chosen clinical description of either schizophrenia (“Appendix 1”) or depression (“Appendix 2”). The questionnaire was self-administered either in the presence of the researcher at the parish church or in his/her absence, according to the participant’s preference. Information on priests’ demographic variables (age and level of education), professional background (duration in the priesthood; years lived in the jurisdiction; role in the parish church; commitment in other activities, such as social or teaching activities, in addition to ecclesiastical function) and experience with persons with MDs (knowledge of persons with MDs among churchgoers, friends, family and priests; reasons why persons with MDs may attend the parish) were also collected.

### Assessment Instrument

The OQ-priest is a self-reported tool available in Italian. The tool includes items from the OQ and ad hoc added items to specifically investigate the study context. From the OQ previously validated questionnaire (Magliano et al. 2017a, 2018), items of the following sections were used: (a) causal beliefs section: sixteen yes/no items grouped into four factors: biological, substance-abuse, stress-related and traumatic causes of MD; (b) recommended professionals section: four yes/no items on health professionals to be involved in the treatment of persons with MD; (c) psychosocial consequences section: seventeen items grouped into the following factors: possibility of recovery, the usefulness of pharmacological and psychological therapies, need for long-term pharmacological therapies, the insight of persons with MDs, perception of social distance from persons with MDs and dangerousness, difficulties of persons with MDs in having romantic relationships. Section c items were rated on a 3-point scale from 1 = “*not true*” to 3 = “*completely true*”. Furthermore, four causes were added to section *a* (wrong therapy, frequenting bad company, possession, and magic), and two items to section *b* (priest and exorcist as recommended professionals). Moreover, 12 items were added to section *c* that were potentially groupable into five subscales: recognizability of persons with MDs, usefulness of prayer and exorcism as therapies for MDs, parenting skills of persons with MDs, opportunity for persons with MDs to be involved in sacraments and parish activities, and behaviors to be adopted with these persons during religious celebrations (same rating scale as for OQ section *c* items). In this study, the psychometric properties of the OQ-priest version have been tested using confirmatory factor analysis (CFA) and Cronbach’s α coefficient (see Statistical Analyses, Results sections and Tables 1, 2).

**Table 1:** Priest socio-demographic variables, professional characteristics and contact with persons with MDs (total sample, *N* = 559)

Age, mean ± SD	52.7 ± 13.1
Education, *N* (%)	
Secondary school degree	6 (1.1)
Bachelor degree	450 (80.5)
Master’s degree or Ph.D. in Theology	103 (18.4)
Years lived in the jurisdiction, mean ± SD	30.5 ± 21.3
Duration in the priesthood, mean ± SD	22.6 ± 14.4
Role in the parish church, *N* (%)	
Parish priest	463 (82.8)
Auxiliary parish priest	96 (17.2)
Knowledge of persons with MDs attending the parish church, yes, *N* (%)	519 (92.8)
Reasons that persons with MDs attended the parish church, *N* (%)	
Attending celebrations	313 (57.7)
Participating in religious group	156 (28.8)
Participation in voluntary activities	89 (16.4)
Receiving individual support	301 (55.5)
Receiving economic help	168 (31.0)
Receiving advices	285 (52.6)
Attending rituals aimed to solve the MD	39 (7.2)
Other reasons	26 (7.2)

**Table 2:** Priests’ views of factors involved in the development of schizophrenia and depression (total sample, *N* = 558)

Causes	*N*	(%)
Traumatic events	312	55.9
Family conflicts	291	52.2
Stress	285	51.1
Psychological violence	253	45.3
Bereavements	246	44.1
Disillusionment in love	232	41.6
Use of street drugs	211	37.8
Work difficulties	209	37.5
Poor parenthood	182	32.6
Sexual abuses	181	32.4
Use of alcohol	179	32.1
Physical violence	172	30.8
Heredity	165	29.6
Physical illness	135	24.2
Magic	101	18.1
Frequenting bad company	87	15.6
Illness in pregnancy	73	13.1
Wrong therapy	68	12.2
Chemical imbalance	64	11.5
Possession	57	10.2

One case missing for causes’ section

### Statistical Analyses

CFA was performed on the global sample to verify the construct validity of the OQ-priest version, section *a* items and section *c* items, respectively. Maximum likelihood estimation of the covariances was applied. Cronbach’s α values were computed to explore the content validity of the confirmed factors in each CFA. *χ*^2^ was used to compare the two groups (schizophrenia and depression) in their views about professionals to be involved in the care of persons with MD (OQ section *b*). The median of the priests’ age was used to split the total sample into a group of younger priests and a group of older priests. Multivariate analysis of variance (MANOVA) was performed to compare the mean scores of causal factors (OQ section *a* modified—dependent variables) among the two diagnostic groups (schizophrenia and depression—independent variables) and the two priest age groups (younger priest group and older priest group—independent variables). The same analysis was performed to compare the mean scores of psychosocial consequences factors (OQ section *c* modified—dependent variables) among the two diagnostic groups (schizophrenia and depression—independent variables) and the two priest age groups (younger priest group and older priest group—independent variables). In both MANOVAs, interactions between the independent variables were also examined. The statistical significance level was set at *p *< 0.05. CFAs were performed using the LISREL 8 Program (Jöreskog and Sörbom 1996). Other tests were performed using the SPSS package, version 21 (IBM 2012).

## Results

### Participant Characteristics

Of the 609 priests approached, 559 (91.8%) agreed to participate and completed the questionnaire (reasons for non-participation: lack of time and/or not interested, 33; disagreement with research aims, 4; no opinion regarding persons with MD, 2; unknown, 5; unwilling to give such information to people outside the religious organization, 6). The socio-demographic and professional characteristics of the 559 participating priests are reported in Table 1. Participants had a mean age and a median age of 52.7 (±13.1 SD) and 51.0 years, respectively. Most priests (80.5%) had a bachelor’s degree in theology. The 559 priests served 173 out of the 181 (95.6%) inhabited areas included in the selected religious jurisdictions. Participants had a mean duration in the priesthood of 22.6 ± 14.4 years and had lived on the jurisdiction area for 30.5 ± 21.3 years. Among them, 82.8% (463) were parish priest and 17.2% (*N* = 96) were auxiliary parish priests. Nine-three percent of priests reported that persons with MDs attended the parish church, for reasons such as: participating in celebrations (57.7%), religious groups (28.8%) and voluntary activities (16.4%); and, receiving individual spiritual support (55.5%), advices (52.6%), and economic help (31%). Of the 559 respondents, 282 completed the OQ-priest after reading a clinical description of schizophrenia and 277 completed the same questionnaire after reading a clinical description of depression. Com’era prima: The two groups were comparable in all variables listed above except for the priests’ contact with persons with MDs, which was higher in the depression group than in the schizophrenia group (90.1% vs. 96.8%, *χ*^2^ 10.01, *df* 1, *p *< 0.001). Furthermore, the number of respondents stating that persons with MDs attended the parish church to receive spiritual help and advices (48.4% vs. 62.9%, *χ*^2^ 11.6, *df* 1, *p* < 0.001; 45.8% vs. 59.6%, *χ*^2^ 10.2, *df* 1, *p* < 0.001) and to attend religious groups and voluntary activities (21.1% vs. 36.7%, *χ*^2^ 16.1, *df* 1, *p* < 0.0001; 10.2% vs. 22.8%, *χ*^2^ 15.8, *df* 1, *p* < 0.0001) was higher in the depression group.

### Descriptive Results of the Total Sample

In the total sample of 558 (1 missing in causal beliefs section) priests, the factors most frequently reported as involved in the development of the MD were traumatic events (55.9%), family conflicts (52.2%) and stress (51.1%; Table 2). Heredity was reported by 29,6% and chemical imbalance by 11.5% of the respondents. Magic and evil possession were reported by 18.1% and 10.2% of the priests, respectively. Seventy percent of priests recommended a psychologist and 52.9% a psychiatrist among the treating professionals. Moreover, 26.9% recommended a neurologist and 12.5% a general practitioner. Furthermore, 44.1% suggested the involvement of a priest and 7.2% an exorcist.

In the total sample of 559 priests, 57.7% of respondents thought it was “completely true” that persons with MD could recover, and 10.7% were equally sure that people with MD should take drugs for life (Table 3). Fifty-seven percent of respondents were convinced that psychological interventions are useful for the treatment of MD and 45.2% were sure of the usefulness of prayer. Forty-five percent of priests thought that people does not know how to behave with persons with MD, while 29.3% thought that these persons are kept at a distance by the others. Thirty percent of participants were completely sure that these persons are unpredictable, while 12.7% of priests were completely sure that persons with MD are dangerous to others. Fifty-six percent fully agreed that persons with MD could be witness in the sacraments, and 52.2% fully agreed that these persons who have recovered could be responsible for parish activities. As far as the behaviors toward people with MD to be adopted during celebrations, 4.4% of priest firmly believed that these persons should be separated from other churchgoers and 6.4% firmly believed that persons with MD should be supervised.

**Table 3:** Priests’ views of persons with schizophrenia and depression (total sample, *N* = 559)

Items	Not true		Partially true		Completely true	
	*N*	(%)	*N*	(%)	*N*	(%)
§ Can recover	26	5.0	196	37.3	303	57.7
Drugs are useful for §	40	7.7	320	61.7	159	30.6
Psychological interventions are useful for §	10	1.8	223	40.9	312	57.2
Prayer is useful for §	31	5.7	266	49.1	245	45.2
Exorcism is useful for §	242	52.4	178	38.5	42	9.1
§ Must take drugs over the life	246	54.8	155	34.5	48	10.7
If stop taking drugs, § become dangerous	156	34.6	221	49.0	74	16.4
If stop taking drugs, § become unwell again	87	19.1	251	55.2	117	25.7
§ Are unpredictable	74	14.5	281	55.1	155	30.4
§ Are kept at distance by the others	108	19.9	275	50.6	161	29.6
People does not know how to behave with §	46	8.5	253	46.7	243	44.8
People does not understand the difficulties experienced by §	47	8.7	262	48.3	233	43.0
People is frightened by §	97	18.3	266	50.1	168	31.6
§ Are dangerous to themselves	63	12.4	343	67.5	102	20.1
§ Are dangerous to others	129	25.5	312	61.8	64	12.7
It is difficult for § to have a love relationship	192	38.5	210	42.1	97	19.4
It is difficult for § to get married or to live together with a partner	179	35.0	200	39.1	132	25.8
§ Who are now well, may be responsible for parish activities (e.g., catechesis, etc.)	46	8.8	204	39.0	273	52.2
§ Are reliable when they confess	52	10.7	290	59.7	144	29.6
§ Can be witnesses in the sacraments (e.g., marriage)	66	12.7	162	31.2	292	56.2
§ Can receive the Eucharist	17	3.1	78	14.1	459	82.9
During religious celebrations, § create discomfort to other churchgoers	278	52.2	219	41.1	36	6.8
During religious celebrations, § should be separated from other churchgoers	457	83.2	68	12.4	24	4.4
During religious celebrations, § should be supervised	318	58.1	194	35.5	35	6.4
§ Are recognizable	142	27.0	257	49.0	126	24.0
§ Do not realize that they are ill	57	10.9	308	58.9	158	30.2
§ Do not realize when they become unwell	67	13.1	263	51.3	183	35.7

§ Persons with a condition like that reported in the clinical description

### Confirmatory Factor Analyses (CFA)

CFA performed on the 20 causes included in section *a* of the questionnaire, confirmed a five-factor structure. The final model fits the data well, *χ*^2^(558) = 341.86, *df* 160, *p *< 0.001; non-normed fit index [NNFI] = 0.93; comparative fit index [CFI] = 0.94; root mean square error of approximation [RMSEA] = 0.04, 90% CI (0.04; 0.05); standardized root mean square residual [SRMR] = 0.05. All factor loadings were significant at the *p* < 0.001 level. Cronbach’s *α* values ranged from 0.48 to 0.75. The details of the psychometric analyses for section *a* are presented in Table 4.

**Table 4:** Confirmatory factor analysis on the QO section a items (*N* = 558)

Items	Factor loadings				
	1	2	3	4	5
Physical violence	0.31				
Psychological violence	0.26				
Traumatic events	0.18				
Sexual abuses	0.31				
Poor parenthood	0.21				
Heredity		0.10			
Chemical imbalance		0.13			
Illness in pregnancy		0.14			
Physical illness		0.18			
Wrong therapy		0.18			
Use of alcohol			0.38		
Use of street drugs			0.36		
Possession				0.15	
Frequenting bad company				0.19	
Magic				0.22	
Family conflicts					0.20
Work difficulties					0.26
Stress					0.27
Bereavements					0.25
Disillusionment in love					0.26
Cronbach’s *α*	0.65	0.48	0.75	0.54	0.63

Factor 1: traumatic causes; 2: biogenetic causes; 3: use of alcohol and street drugs; 4: supernatural causes; 5: stress-related causes

The correlations among the five factors were all significant for *p* < 0.01 (standardized solution)

CFA performed on the 29 items included in section *c* of the OQ-priest version confirmed the 13-factor structure listed above, with the exclusion of one item. The final model fits the data well, *χ*^2^(559) = 559.13, *df* 252 *p *< 0.05; non-normed fit index [NNFI] = 0.91; comparative fit index [CFI] = 0.93; root mean square error of approximation [RMSEA] = 0.05, 90% CI (0.04; 0.05); standardized root mean square residual [SRMR] = 0.05. All factor loadings were significant at the *p* < 0.001 level. Cronbach’s *α* values ranged from 0.52 to 0.84. The details of the psychometric analyses for section *c* are presented in Table 5.

**Table 5:** Confirmatory factor analysis on the QO section c items (*N* = 559)

Items	Factor loadings												
	1	2	3	4	5	6	7	8	9	10	11	12	13
§ Can recover	0.57												
Drugs are useful for §		0.55											
Psychological interventions are useful for §			0.53										
Prayer is useful for §				0.59									
Exorcism is useful for §					0.59								
§ Must take drugs over the life						0.40							
If stop taking drugs, § become dangerous						0.46							
If stop taking drugs, § become unwell again						0.38							
§ Are unpredictable							0.23						
§ Are kept at distance by the others							0.43						
People does not know how to behave with §							0.43						
People does not understand the difficulties experienced by §							0.41						
People is frightened by §							0.47						
§ Are dangerous to themselves								0.29					
§ Are dangerous to others								0.54					
It is difficult for § to have a love relationship									0.55				
It is difficult for § to get married or to live together with a partner									0.66				
§ Who are now well, may be responsible for parish activities (e.g., catechesis, etc.).										0.27			
§ Are reliable when they confess										0.22			
§ Can be witnesses in the sacraments (e.g., marriage)										0.39			
§ Can receive the Eucharist										0.20			
During religious celebrations, § create discomfort to other churchgoers											0.44		
During religious celebrations, § should be separated from other churchgoers											0.34		
During religious celebrations, § should be supervised											0.46		
§ Are easily recognizable												0.69	
§ Do not realize that they are ill													0.39
§ Do not realize when they become unwell													0.32
Cronbach’s *α*	–	–	–	–	–	0.75	0.75	0.68	0.84	0.55	0.77	–	0.52

§ PWC = Persons With a Condition like that reported in the description; Factor 1: possibility to recover; 2: usefulness of pharmacological treatments; 3: usefulness of psychological therapies; 4: usefulness of prayer; 5: usefulness of exorcism; 6: need of long-term pharmacological therapies; 7: perception of social distance from PWC; 8: perception of dangerousness; 9: difficulties of PWC in affective relationships; 10: participation of PWC in parish activities and sacraments; 11: discriminatory behaviors in religious celebrations; 12: recognizability of PWC; 13: insight of PWC. The correlations among the thirteen factors were all significant for *p* < 0.05, except those between factor 1 with 2, 7; between factor 2 and 4, 7, 8, 9, 10, 12 and 13; between factor 3 and 5, 6, 7, 8, 9, 10, 12 and 13; between factor 4 and 7, 8, 9, 10, 12 and 13; between factor 5 and 7, 9, 12 and 13; between factor 7 and 10; between factor 12 and 13 (standardized solution)

### Differences in Priests’ Views Between the Two Groups

MANOVA performed on causal factors 1–5 of the OQ-priest section *a* revealed a significant effect of the diagnostic group on two of the factors (Wilks’ *λ* = 0.93, *F* (5, 550) = 7.88, *p *< 0.0001; Table 6). Otherwise, neither the priests’ age (Wilks’ *λ* = 0.98, *F* (5, 550) = 2.16, *p *< 0.05) nor the interaction between the diagnostic group and the priests’ age had any effect on the causal factors (Wilks’ *λ* = 0.98, *F* (5, 550) = 2.36, *p *< 0.05; no factor reached minimum level of accepted significance). In particular, MANOVA showed that in the schizophrenia group, priests reported less traumatic factors and stress-related factors among the causes of the disorder compared to the depression group. Regarding the professionals to be involved in the care of MD, in the schizophrenia group, a higher percentage of respondents recommended psychiatrists (59.2% vs. 46.4%, *χ*^2^ = 9.2, *df* 1, *p *< 0.002) and *not* recommended psychologists (65.2% vs. 75.4%, *χ*^2^ = 6.8, *df* 1, *p *< 0.009) and priests (37.9% vs. 50.4%, *χ*^2^ = 8.7, *df* 1, *p *< 0.003) among the treating professionals.

**Table 6:** Priests’ views on causes of MDs: differences between diagnostic groups

Casual factors*	Diagnostic groups		MANOVA *F* (1, 557)
	Schizophrenia (*N* = 281)	Depression (*N* = 277)	
	Mean ± SD	Mean ± SD	
Traumatic	0.35 ± 0.29	0.44 ± 0.32	9.80^a^
Stress-related	0.37 ± 0.30	0.53 ± 0.31	35.9^b^
Biogenetic	0.17 ± 0.21	0.19 ± 0.22	0.35
Substances’ misuse	0.33 ± 0.42	0.37 ± 0.43	1.20
Supernatural	0.13 ± 0.24	0.16 ± 0.43	1.65

^a^*p *< 0.005; ^b^*p *< 0.0001

MANOVA performed on factors 1–13 of the OQ-priest section *c* revealed a significant effect of the diagnostic group on five of the factors (Wilks’ *λ* = 0.95, *F* (13, 543) = 1.99, *p *< 0.02; Table 7) and of the priests’ age on five factors (Wilks’ *λ* = 0.91, *F* (13, 547) = 3.97, *p *< 0.0001). No significant interaction between the two independent variables was found (Wilks’ *λ* = 0.98, *F* (13, 547) = 0.98, *p *> 0.05). In particular, MANOVA highlighted that in the schizophrenia group, priests were less optimistic about the possibility of recovery compared to the depression group. In the schizophrenia group, priests were surer of the need for long-term pharmacological treatments and more skeptical about the usefulness of prayer in the care of the disorder. Furthermore, in the schizophrenia group, priests were more convinced of the difficulties experienced by persons with MD in affective relationships and more doubtful regarding these persons participating of in parish activities and sacraments. As far as the effect of priests’ age on priests’ opinions, compared to the younger priests, the older priests acknowledged more relevance to prayer in the care of MDs and they were more convinced of the need for long-term pharmacological treatments. Moreover, the older priests were surer that persons with MDs had difficulties in affective relationships, were recognizable and were kept at distance by the others.

**Table 7:** Priests’ views on treatments and psychosocial consequences of MDs: differences between diagnostic groups and priests’ age groups

	Diagnostic groups		MANOVA *F* (1, 558)	Priests’ age groups		MANOVA *F* (1, 558)
	Schizophrenia (*N* = 282)	Depression (*N* = 277)		Priests’ age ≤ 51 years (N = 282)	Priests’ age > 51 years (N = 277)	
	Mean ± SD	Mean ± SD		Mean ± SD	Mean ± SD	
Possibility to recover	2.46 ± 0.55	2.60 ± 0.58	7.94^c^	2.57 ± 0.57	2.49 ± 0.57	2.63
Usefulness of pharmacological treatments	2.27 ± 0.56	2.20 ± 0.54	2.78	2.23 ± 0.54	2.23 ± 0.58	0.009
Usefulness of psychological therapies	2.56 ± 0.52	2.55 ± 0.53	0.11	2.54 ± 0.52	2.57 ± 0.54	0.50
Usefulness of prayer	2.35 ± 0.60	2.44 ± 0.57	4.26^a^	2.33 ± 0.56	2.47 ± 0.60	8.46^b^
Usefulness of exorcism	1.55 ± 0.57	1.59 ± 0.62	0.73	1.53 ± 0.55	1.60 ± 0.64	1.90
Need of long-term pharmacological therapies	1.86 ± 0.48	1.76 ± 0.49	5.42^a^	1.74 ± 0.46	1.89 ± 0.50	13.08^d^
Perception of social distance from PWC	2.24 ± 0.41	2.19 ± 0.49	1.63	2.16 ± 0.44	2.28 ± 0.46	8.53^b^
Perception of dangerousness	1.98 ± 0.49	1.97 ± 0.47	0.02	1.95 ± 0.46	2.0 ± 0.50	1.15
Difficulties of PWC in affective relationships	1.93 ± 0.66	1.78 ± 6.55	7.13^b^	1.79 ± 0.65	1.93 ± 0.66	6.67^b^
Participation of PWC in parish activities and sacraments	2.43 ± 0.37	2.50 ± 0.38	4.24^a^	2.48 ± 0.38	2.45 ± 0.38	0.68
Discriminatory behaviors in religious celebrations	1.45 ± 0.48	1.38 ± 0.46	2.98	1.42 ± 0.49	1.41 ± 0.45	0.20
Recognizability of PWC	2.00 ± 0.68	1.93 ± 0.70	1.20	1.82 ± 0.66	2.12 ± 0.69	26.08^d^
Insight of PWC	2.23 ± 0.50	2.18 ± 0.50	1.72	2.21 ± 0.49	2.21 ± 0.51	0.003

*PWC * persons with a condition like that reported in the description

^a^*p *< 0.05; ^b^*p *< 0.01; ^c^*p *< 0.005; ^d^*p* < 0.0001

## Discussion

### Interpretation of the Results

The results of this study show that priests have both similarities and differences in their views of schizophrenia and depression and their attitudes toward persons with these disorders. In both diagnostic groups, priests attributed the disorder mainly to psychosocial causes and gave the lowest importance to supernatural causes. These data are in line with those from studies among Catholic clerics in Lebanon (Aramouny et al. 2020), Muslims and Catholics clergy in Ethiopia (Jacobsson and Merdasa 1991) and among Arabic-speaking religious leaders in Australia (Youssef and Deane 2003). Conversely, these findings are in contrast with the results of other studies carried out among American Methodist pastors (Lafuze et al. 2002), and among Euro-American Presbyterian pastors (Yamada et al. 2018).

The high importance given to psychosocial causes within each diagnostic group is probably due to the priests’ high frequency of contact with people with MDs who attended the parish church with spiritual and material needs. As outlined by Aramouny et al. (2020), priests might be mostly exposed to persons whose mental health problems have been caused or strongly associated with traumatic events and problematic living conditions. In contrast with the stereotypical expectation of the priests as giving a prevalent religious interpretation of MDs, the mean score of the supernatural causes factor was low in both the groups. These results suggest that most priests tend to distinguish mental health problems from circumstances interpreted from a faith perspective (Leavey 2010). The low importance given to supernatural causes, a finding in line with that from the study by Aramouny et al. (2020), is also supported by the low percentage of priests indicating the exorcist among the recommended professionals in both the groups. These results might be partially explained by a shift from Biblical times—when talk of demons and mental torment was not uncommon—to a bold medicalization of mental health problems, particularly schizophrenia, fostered by drug companies (Mosher et al. 2013) and the media (Magliano and Marassi 2018).

While the two diagnostic groups were similar in the relevance given to substance, biological and supernatural causes in the development of the disorder, the groups appear significantly different in the mean score of the stress-related and traumatic causal factors. The lower importance given to psychosocial causes in schizophrenia versus depression may be due to the lower frequency of priests having contact with persons with psychotic symptoms. Sometimes persons diagnosed with schizophrenia tend to exclude themselves in the belief that they are discriminated against (Thornicroft et al. 2009). Another reason for the difference in the relevance of psychosocial causal factors between the two diagnostic groups could be the different social representations of depression and schizophrenia in the media. Depression is frequently presented as due to a wide range of life adversities that can happen to everyone (Magliano et al. 2017b), whereas schizophrenia is frequently presented as a brain disorder that can be exacerbated by the stress (Magliano and Marassi 2018).

As far as the professionals to be involved in the care of MDs, psychologists and priests were more frequently recommended in the depression group, while psychiatrists were more frequently recommended in the schizophrenia group. The greater importance given to traumatic events and stress-related causes in depression can partly explain the high percentage of priests who indicated non-medical professionals for the treatment of this disorder. Beliefs about severity and need for long-term pharmacological interventions in schizophrenia may be a reason for the more frequent recommendation of the expertise of psychiatrists in this diagnostic group (Ellison et al. 2006). It should be highlighted that in nearly all cases, priests recommended religious professionals were in association with health professionals. This finding further confirms that most priests distinguish mental health problems from religious circumstances and view themselves and health professionals as meeting two different needs of believers with MD. However, it should not be excluded that the not very high percentage of priests believing in the healing power of God in schizophrenia and depression is related to the medicalization of MDs (Deacon 2013). As priests stated verbally, their involvement in the treatment of believers with MD should be intended as a source of spiritual support distinct from the health treatments. Priests should be trained on how to approach people with MDs and facilitate trusted relationships between these people and health care professionals, when necessary (Carey and Del Medico 2013). Data of this study suggest that collaboration between priests and mental health professionals is possible, while respecting each other’s competence (Farrell and Goebert 2008).

Regarding the treatments of MD, in line with data from previous studies (Aramouny et al. 2020; Youssef and Deane 2003), priests value psychosocial interventions as more useful than pharmacological treatments. Moreover, no difference was found in the usefulness acknowledged for health treatments in schizophrenia and depression. This result may be important for the advices about therapies that priests give to believers with MDs. This is even more relevant in the case of schizophrenia, where treatments are unbalanced in favor of drug treatments, and low importance is given to evidence-based psychological treatments (Ince et al. 2016). The relevance given to mental health professionals and psychological therapies may have been influenced by an interview given by the Pope Francis to a French sociologist in 2017. In the interview that had great media resonance in Italy, the Pope referred to his personal experience of psychotherapy stating “I, too, who am Pope, needed help. I searched for it and benefited from it. Trust me. Get help” (cited by Carpiniello 2017). Priests are more skeptical regarding recovery in schizophrenia than in depression. The greater prognostic optimism regarding depression may be related to greater social acceptance of this disorder sometimes viewed as a transient experience that can affect everyone over the course of life (Munizza et al. 2013; Magliano et al. 2017b). Conversely, schizophrenia is often presented as an incurable brain disease requiring life-long pharmacological treatments to be controlled (Magliano and Marassi 2018b; Read et al. 2013; Kvaale et al. 2013). Skepticism about the possibility of recovery in schizophrenia can adversely affect the hope priests may convey to churchgoers with psychotic symptoms (Heffernan et al. 2016) and it may discourage persons with these disorders from making efforts to improve their quality of life.

We found no difference in the level of perceived dangerousness and social distance from persons with MD between schizophrenia and depression. However, it is not to be excluded that priests’ responses to the items of these subscales would have been influenced by social desirability bias. As reported by Latkin et al. (2017), “social desirability bias is the tendency to underreport socially undesirable attitudes and behaviors and to over report more desirable attributes”. Sometimes, socially desirable response might be explained by conscious and unconscious desires to avoid embarrassment (Tourangeau and Yan 2007). The priest does not wish to appear stigmatizing toward persons with MDs, particularly those with schizophrenia. Such a situation would lead the priest to identify the dangerousness in the perception of the others, as supported by the correlation between the subscales of perception of social distance by the others and of perception of dangerousness by the respondents (*r* = 0.30, *p* < 0.0001; *r* = 0.32, *p* < 0.0001).

The results of this study also showed that compared to the younger priests, the older priests more firmly believed that persons with MDs have difficulties in affective relationships, are recognizable and kept at the distance by the others. These findings are in line with those from general population studies showing that negative attitudes and desire of social distance from persons with MDs are greater at older ages (Jorm and Oh 2009).

Priests considered persons with schizophrenia as less capable of participating in parish activities and sacraments than those with depression. It is likely that these differences may in part reflect the messages conveyed by the media contributing to social acceptance of persons with depression and to discrimination against those with schizophrenia (Magliano and Marassi 2018). Priests’ reluctance regarding the involvement of persons with schizophrenia in religious activities may hinder connections of persons with these disorders with other churchgoers, further increasing the exclusion of these persons from contexts with potential rehabilitative effects. Furthermore, denying people with mental disorders access to pastoral care and the sacraments can adversely affect their mental and spiritual health. These findings highlight that persons with MDs, especially those with schizophrenia, are sometimes exposed to stigma even in religious settings. Educating priests about MDs and sensitizing them regarding the toxic effect of stigma (Magliano et al. 2016) on the lives and recovery processes of persons with MDs can be of dual benefit. On the one hand, it can facilitate the use of mental health services by believers with these disorders (Farrell and Goebert 2008), especially by those refusing to get medical help (Carey and Del Medico 2013). On the other hand, educating priests on stigma, can foster the fulfillment of the spiritual needs of these persons and their participation in the religious community of reference.

### Strengths and Limitations of the Study

This is the first study carried out in Italy that specifically explored Catholic priests’ views of persons with MDs and whether they varied in relation to a clinical description of schizophrenia or depression. Among the strengths of the study are the inclusion of a large sample randomly assigned to the diagnostic groups, the face-to-face data collection, and the high participation rate (92%) suggesting that priests are interested in caring for persons with MD. The use of a self-reported questionnaire adapted to the study context and re-validated is an additional strength, also facilitating its replication in other religious contexts. The study has a number of limitations suggesting caution in the interpretation of its results. In particular, the inclusion of priests only from southern Italy, a geographical area where healthcare resources are poorer (Magliano et al. 2002) is a limitation that might have influenced the priests’ beliefs about treatments and prognosis. The lack of other Italian studies of priests’ beliefs about persons with MDs means we do not know whether our findings are generalizable to other areas of Italy. We are planning to extend the study to other geographical areas of Italy. Another limitation is the lack of a control group on priests’ views of persons with a physical illness which may help in the interpretation of some results, particularly those related to the psychosocial consequences of MDs. This limitation prevents us from establishing how and whether priests’ views of persons with MD are influenced by the stigma associated with these disorders. A further limitation is the lack of data on the participants’ professional background, such as information on where the priests were trained and whether they had received formal training in mental health or psychology as part of their degree. These limitations prevent us from evaluating the potential influence of training on the priests’ opinions. In addition, we did not collect data on priests’ personal experience of MDs. Furthermore, the survey investigated only the *opinions* of priests, and may not reflect their actual behaviors with persons with MDs. However, the fact that almost half of the sample stated that persons with MD attended the own parish church to receive individual spiritual support and advices suggest that priests’ opinions in part reflect the acceptance of these persons in the local religious community. Some of these limitations will be addressed in further studies at their planning stage.

## Data Availability

The data that support the findings of this study are available from the corresponding author upon reasonable request, which must include a protocol and statistical analysis plan and not be in conflict with our publication plan.

## Appendix 1

Some people sometimes seem unable to distinguish between things that really happen and are experienced by other people, and things that happen only in their mind. Sometimes, these people believe or say things that seem bizarre or absurd to other people, or hear voices, smell things, or see images that other people do not. Sometimes, these people may have difficulty expressing their feelings or behaving appropriately (for instance, they may cry in response to a positive event, or may appear happy following an unpleasant one), or they may remain shut up in their house for a long time, or talk very little or not at all. They behave as if they lived in a world of their own, apparently without interest in anything or anybody. Sometimes they may have muddled thoughts, may invent odd or incomprehensible words, may lose the thread of the speech, or they may jump from one issue to another with no apparent reason.

## Appendix 2

Some people sometimes feel sad, down, unable to feel pleasure, or to have interest for those activities they liked in the past. Sometimes, these people feel incompetent, may believe to be derided by the others, and make themselves feel guilty for trivial things. These people may have no hope for future and when their feelings of sadness and worthlessness become unbearable, they may decide to stop living. Sometimes, these people may have difficulties in eating and sleeping regularly, and may feel poor concentrated or physically tired. Other times, they may feel irritable and get annoyed with the others for unimportant things.
